# Impact of Resveratrol on Glucose Control, Hippocampal Structure and Connectivity, and Memory Performance in Patients with Mild Cognitive Impairment

**DOI:** 10.3389/fnins.2017.00105

**Published:** 2017-03-07

**Authors:** Theresa Köbe, A. Veronica Witte, Ariane Schnelle, Valentina A. Tesky, Johannes Pantel, Jan-Philipp Schuchardt, Andreas Hahn, Jens Bohlken, Ulrike Grittner, Agnes Flöel

**Affiliations:** ^1^Department of Neurology, Charité - University Medicine BerlinBerlin, Germany; ^2^NeuroCure Cluster of Excellence, Charité - University Medicine BerlinBerlin, Germany; ^3^Department of Neurology, Max Planck Institute of Human Cognitive and Brain SciencesLeipzig, Germany; ^4^SFB 1052 Obesity Mechanism Subproject A1, University of LeipzigLeipzig, Germany; ^5^Department of General Medicine, Goethe-UniversityFrankfurt am Main, Germany; ^6^Department of Nutrition Physiology and Human Nutrition, Gottfried Wilhelm Leibniz UniversityHannover, Germany; ^7^Medical Practice Bohlken for Neurology and PsychiatryBerlin, Germany; ^8^Biostatistics and Clinical Epidemiology, Charité - University Medicine BerlinBerlin, Germany; ^9^Center for Stroke Research Berlin, Charité - University Medicine BerlinBerlin, Germany; ^10^Department of Neurology, University Medicine GreifswaldGreifswald, Germany

**Keywords:** hippocampus, memory performance, MCI, resting-state functional connectivity, resveratrol

## Abstract

In healthy older adults, resveratrol supplementation has been shown to improve long-term glucose control, resting-state functional connectivity (RSFC) of the hippocampus, and memory function. Here, we aimed to investigate if these beneficial effects extend to individuals at high-risk for dementia, i.e., patients with mild cognitive impairment (MCI). In a randomized, double-blind interventional study, 40 well-characterized patients with MCI (21 females; 50–80 years) completed 26 weeks of resveratrol (200 mg/d; *n* = 18) or placebo (1,015 mg/d olive oil; *n* = 22) intake. Serum levels of glucose, glycated hemoglobin A1c and insulin were determined before and after intervention. Moreover, cerebral magnetic resonance imaging (MRI) (3T) (*n* = 14 vs. 16) was conducted to analyze hippocampus volume, microstructure and RSFC, and neuropsychological testing was conducted to assess learning and memory (primary endpoint) at both time points. In comparison to the control group, resveratrol supplementation resulted in lower glycated hemoglobin A1c concentration with a moderate effect size (ANOVA_RM_ *p* = 0.059, Cohen's *d* = 0.66), higher RSFC between right anterior hippocampus and right angular cortex (*p* < 0.001), and led to a moderate preservation of left anterior hippocampus volume (ANOVA_RM_ *p* = 0.061, Cohen's *d* = 0.68). No significant differences in memory performance emerged between groups. This proof-of-concept study indicates for the first-time that resveratrol intake may reduce glycated hemoglobin A1c, preserves hippocampus volume, and improves hippocampus RSFC in at-risk patients for dementia. Larger trials with longer intervention time should now determine if these benefits can be validated and extended to cognitive function.

## Introduction

The growing incidence of Alzheimer's disease worldwide and the lack of curative pharmacological approaches increase the demand for alternative preventive strategies at earlier disease stages, i.e., in patients with mild cognitive impairment (MCI). Nutrition is now well-recognized as a factor that influences brain structure, functional connectivity, and cognition and may modulate the rate and degree of disease progression (Gustafson et al., 2015; Huhn et al., 2015).

Caloric restriction and administration of caloric restriction mimetics like the polyphenol resveratrol might provide a promising avenue to slow brain atrophy and cognitive decline (Kim et al., 2007; Dal-Pan et al., 2011; Willette et al., 2012). It has been shown that physiological doses of resveratrol are safe and well-tolerated and that the substance is able to penetrate the blood-brain barrier to influence the central nervous system (Turner et al., 2015).

Several studies in mice demonstrated resveratrol-mediated neuroprotective effects on key features of Alzheimer's disease, including decreased amyloid deposition, reduced tau hyperphosphorylation (Porquet et al., 2013), enhanced neurogenesis in the hippocampus (HC) (Harada et al., 2011), and improved memory function linked to activation of longevity genes, i.e., Silent Information Regulator T1 (Zhao et al., 2013). In primates, spatial memory performance was improved after resveratrol intake (Dal-Pan et al., 2011).

Moreover, first resveratrol interventional trials were conducted in humans, showing positive influence on cerebral blood flow (Kennedy et al., 2010), glucose control (Brasnyo et al., 2011; Bhatt et al., 2012; Crandall et al., 2012), and verbal episodic memory performance (Witte et al., 2014). No beneficial effects on brain volume have been found in human trials so far (Witte et al., 2014; Turner et al., 2015). In sum, beneficial effects in healthy adults are not yet conclusive (Wong et al., 2013; Witte et al., 2014; Wightman et al., 2015), possibly due to short intervention times of 4–6 weeks in trials with a negative outcome (Wong et al., 2013; Wightman et al., 2015). Moreover, studies that investigate the impact of resveratrol on cognition and brain networks in individuals at high-risk for dementia, like MCI patients, are missing so far.

The previous positive trial on resveratrol supplementation in healthy older adults evaluated a 26-week intervention with 200 mg resveratrol, showing an improvement in verbal episodic memory performance. Potential mechanisms underlying this effect were also described, such as a significant decrease in glycated hemoglobin A1c (HbA1c) and an increase in resting-state functional connectivity (RSFC) between the HC and frontal, parietal, and occipital brain regions (Witte et al., 2014).

Based on these findings, we now aimed to improve verbal episodic memory function in memory-impaired patients at high-risk for dementia (primary endpoint). Therefore, we conducted a similarly designed proof-of-concept study (double-blind, randomized-controlled) with MCI patients, assessing the effects of a 26-week resveratrol supplementation. Moreover, we aimed to investigate glucose metabolism, total gray matter volume, and RSFC and structure of the HC, a key region implicated in memory function (Wittenberg and Tsien, 2002) and known to be affected early in the course of Alzheimer's disease (Ries et al., 2008).

## Materials and methods

The study was approved by the Ethics Committee of the Charité University Hospital Berlin, Germany, and was in accordance with the declaration of Helsinki. All subjects provided informed written consent before participation in the study and received a small reimbursement at the end.

### Study participants

Patients (aged 50–80 years) with MCI were recruited in Berlin (Memory Clinic of the Department of Neurology of the Charité University Hospital and Neurology specialist practice) and Frankfurt am Main (Institute of General Practice), Germany. MCI patients (amnestic; single and multiple domain) were diagnosed according to Mayo criteria within 12 months before baseline visit. These criteria comprised a subjective cognitive complaint and an objective memory impairment in standardized tests [performing at least one standard deviation below age- and education-specific norm in relevant subtests of the CERAD-Plus or Rey Auditory Verbal Learning Test (AVLT) battery (Total Word List, Delayed Recall Word List/Figures) (Morris et al., 1989)]. Moreover, patients showed relatively preserved general cognition, no impairment in activities of daily living, and no dementia (Petersen et al., 1999). Exclusion criteria comprised MMSE scores <24 at baseline visit, severe untreated medical, neurological or psychiatric diseases and brain pathologies identified in the magnetic resonance imaging (MRI) scan, no right-handedness (Oldfield, 1971), non-fluent German language and BMI <18 kg/m^2^ or >35 kg/m^2^.

### Study design

**Figure 1:**
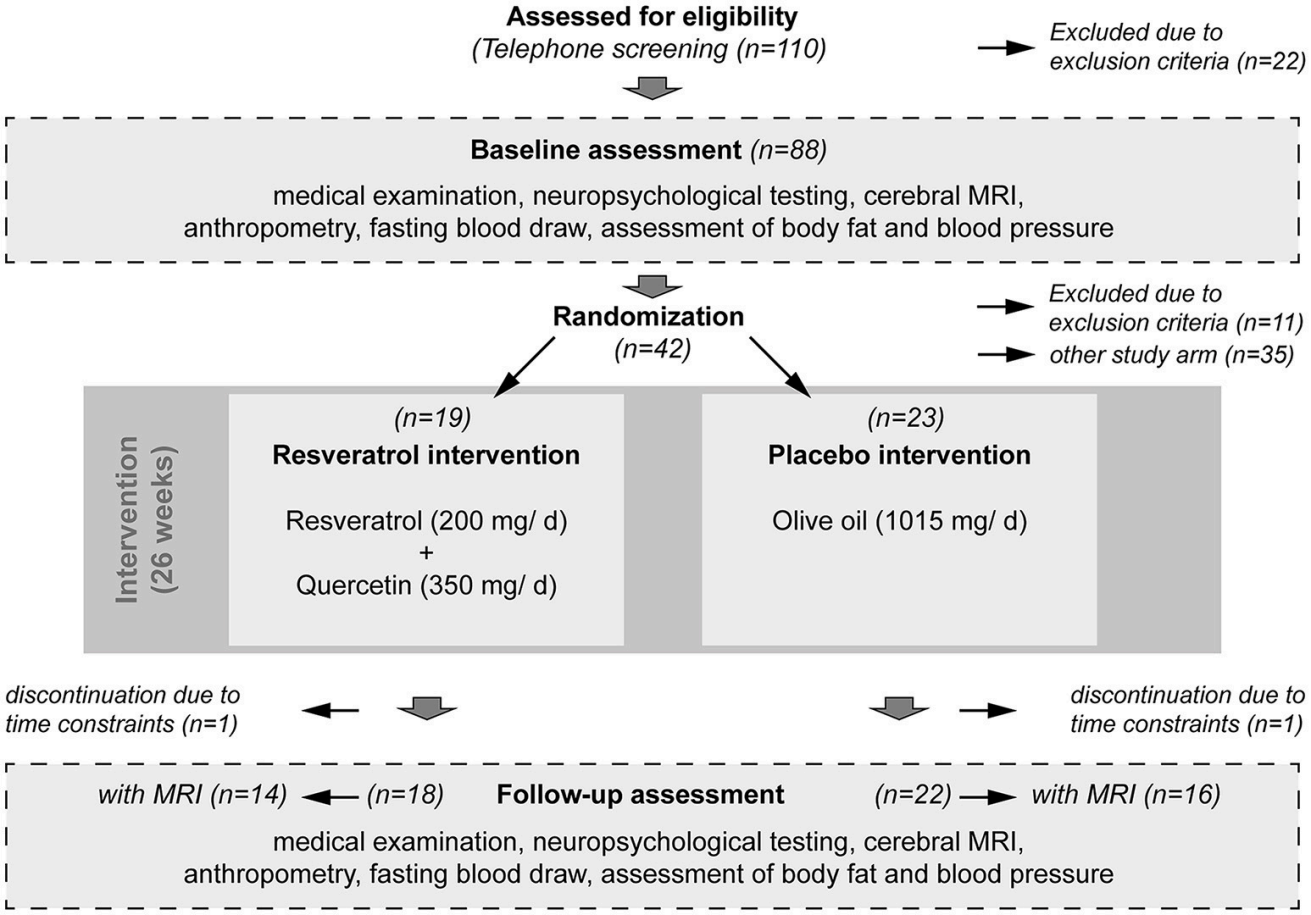
**Study flow chart**. In total, 110 MCI patients were screened on telephone, of which 88 were invited for baseline assessment. Forty-two patients met the inclusion criteria and were randomized to the resveratrol intervention group (*n* = 19) or to the placebo intervention group (*n* = 23). Two patients did not complete participation due to time constraints. Thus, 40 patients successfully completed the intervention over 26 weeks (resveratrol *n* = 18; placebo *n* = 22). Before and after the intervention period, patients underwent a standardized medical examination, including neuropsychological testing, cerebral magnetic resonance imaging (MRI), anthropometry, and fasting blood draw for detection of serum parameters and APOE e4 status. MRI scans from both time points baseline and follow-up were not available for 10 patients (scheduling problems at follow-up), leaving 30 patients for longitudinal MRI analysis (resveratrol *n* = 14; placebo *n* = 16).

One-hundred-ten patients, previously diagnosed with MCI, were screened for study eligibility by telephone (e.g., for additional severe diseases, known brain pathologies, MR ineligibility). Twenty-two had to be excluded on the basis of these criteria. The remaining 88 patients were invited for baseline assessment. From this group, 11 patients had to be excluded either due to a pathological MRI finding (*n* = 3) or due to comorbidities (*n* = 8; Parkinson's disease, depression). Eligible patients were allocated in different intervention groups (details below) by a simple randomization approach, based on a computer-generated list of random numbers, carried out by an investigator that was not involved in the study. Thirty-five patients were randomized into a separate study testing the effect of omega-3 fatty acids on brain structure and function. Two patients (resveratrol *n* = 1; placebo *n* = 1) did not complete the intervention due to time constraints. In total, 40 MCI patients completed the current study (resveratrol *n* = 18; placebo *n* = 22; see Figure 1). MRI scans from follow-up were not available for 10 patients (scheduling problems at follow-up), leaving 30 patients for longitudinal MRI analysis (resveratrol *n* = 14; placebo *n* = 16).

During baseline assessment, patients underwent a standardized medical examination, neuropsychological testing, structural and resting-state functional MRI of the brain, as well as fasting blood sampling, and assessments of anthropometric data and mood (baseline assessment; see Figure 1 and Table 1).

**Table 1:** **Baseline characteristics of MCI patients dependent on group**.

**Characteristic parameters**	**Resveratrol**	**Placebo**
*n* (Women) [*n*]	18 (10)	22 (11)
Age [years]	65 ± 9 (50–80)	69 ± 7 (51–79)
Education [years]	14 ± 4 (8–20)	16 ± 3 (11–19)
Body mass index (BMI) [kg/m^2^]	26 ± 3 (20–31)	26 ± 3 (23–32)
Right-handedness [%]^a^	90 ± 11 (70–100)	81 ± 37 (−50–100)
Systolic blood pressure [mm Hg]^a^	140 ± 20 (117–177)	145 ± 14 (115–168)
LDL-to-HDL ratio	2.5 ± 0.8 (1.4–4.2)	2.2 ± 0.7 (0.9–3.7)
hsCRP [pg/ml]^c^	1.3 ± 2.2 (0.3–9.3)	2.1 ± 1.9 (0.3–7.9)
Smoking [pack years]^a^	11 ± 14 (0–38)	4 ± 8 (0–30)
Physical activity [kcal/week]^a^	3,734 ± 3,844 (321–13,034)	3,199 ± 2,486 (435–9,295)
Beck's Depression Index (BDI) [score]^b^	9 ± 6 (0–20)	9 ± 6 (0–20)
State-Trait Anxiety Inventory-X1 [score]	34 ± 6 (23–45)	39 ± 9 (22–56)
Mini mental state examination (MMSE) [score]	28 ± 2 (24–30)	29 ± 1 (26–30)
Apolipoprotein_rs429358/rs7412 [n e4−; e4+]	13; 5	9; 13

*Data expressed as mean ± SD (range; min-max)*.

a Twelve or

b *four patients were excluded due to missing values, respectively*.

c *One extreme outlier (27.7 pg/ml) within the resveratrol group was excluded (indicating an acute infection). Adjusting for differences in CRP value did not attenuate the beneficial effect of resveratrol on HbA1c concentration, HC RSFC, and HC volume compared with placebo*.

MCI patients were randomized in either a resveratrol or placebo group, receiving a daily supplementation of 200 mg resveratrol, plus 350 mg quercetin to increase bioavailability of resveratrol (De Santi et al., 2000), or 1,015 mg olive oil, respectively. Patients were instructed to follow a regular intake once a day (4 capsules) before or at a main meal for 26 weeks. Resveratrol intake was well tolerated with no reported treatment-related serious adverse events that would have led to study termination. Each patient received half of their study medication at baseline and the second half after 13 weeks.

Capsules were provided by VIA Vitamine, Oberhausen, Germany. Following the intervention, baseline measurements were repeated (follow-up assessment; see Figure 1).

### Compliance of capsule intake

The number of remaining resveratrol or placebo capsules was counted after 13 and 26 weeks, showing a >83% compliance rate. Moreover, patients completed a questionnaire on capsule intake at the end of the study. Misses of capsule intake exceeding 5 times/ month would have led to pre-specified exclusion from analysis. However, none of the patients had to be excluded in this study due to capsule misses. Patients were instructed not to change their dietary habits and their physical activity throughout the intervention.

### Neuropsychological assessment

Learning and episodic declarative memory performance of MCI patients was tested, using the German version of the Rey Auditory Verbal Learning Test (AVLT) (Lezak, 2004). Patients were asked to learn a list of 15 words within five immediate recall trials, followed by a 30 min delayed recall and delayed recognition test. Learning ability was defined as the sum of words learned in all five trials (maximum 75 words). Delayed recall represented the total number of remembered words after 30 min (maximum 15 words). For delayed recognition, MCI patients were asked to recognize the 15 original words presented within 35 distractor words subsequent to the delayed recall test (number of correctly recognized words minus false positive words; maximum 15 words). Trained staff members conducted all tests according to standardized procedures.

### Magnetic resonance image (MRI) acquisition

MRI scanning was conducted at baseline and follow-up, using a 3-Tesla Siemens Trio system with a 12-channel head coil at the Berlin Center for Advanced Neuroimaging.

High resolution T1-weighted scans (3D Magnetization Prepared Rapid Acquisition with Gradient Echoes (MPRAGE); *TR* = 1,900 ms, *TE* = 2.52 ms, 192 sagittal slices, voxel-size of 1.0 × 1.0 × 1.0 mm^3^, flip angle = 9°), and diffusion-weighted spin-echo echo-planar imaging (EPI) scans (*TR* = 7,500 ms, *TE* = 86 ms, 61 axial slices, voxel size of 2.3 × 2.3 × 2.3 mm^3^; 64 directions with a *b*-value of 1,000 s/mm^2^ and 10 b0) were acquired.

Functional scans were obtained at rest using a T2^*^-weighted EPI sequence (*TR* = 2,300 ms, *TE* = 30 ms, 34 slices, voxel size of 3.0 × 3.0 × 4.0 mm^3^, flip angle = 90°). Patients were instructed to keep their eyes closed, relax, think of nothing in particular and move as little as possible during this 6 min scan.

Image preprocessing and analyses were done using the software package FSL 4.1 (http://www.fmrib.ox.ac.uk/fsl), AFNI 2011 (http://afni.nimh.nih.gov/afni), and FreeSurfer 5.3 (http://surfer.nmr.mgh.harvard.edu/), as indicated below.

### MRI analyses

MRI analyses were performed according to Witte et al. (2014), where the procedure is described in detail.

Briefly, volumetric delineation of the total gray matter, and left and right HC was conducted, using FSL and Freesurfer brain extraction tools (BET and mri_watershed), FMRIB's Automated Segmentation Tool (FAST) and Integrated Registration and Segmentation Tool (FIRST). To determine the respective anterior and posterior parts, the center of gravity of the HC was assessed after rigid body transformation to MNI space and the corresponding y-coordinate then served as a measure for individual anterior/posterior-division.

Individual HC volumes were adjusted for intracranial volume (ICV), according to previous studies (Raz et al., 2005; den Heijer et al., 2012; Kerti et al., 2013), using the following formula: adjusted volume = raw volume − b × (ICV − mean ICV). The coefficient b represents the slope of regression of a region of interest volume on ICV. The results of HC segmentation were superimposed on anatomic images and visually inspected to exclude misregistration or erroneous HC identification.

Hippocampal microstructure was assessed by mean diffusivity (MD), estimated by using diffusion tensor imaging (DTI), in line with previous studies (den Heijer et al., 2012; Kerti et al., 2013). Therefore, a tensor model was fitted to the motion-corrected DTI data at each voxel to create individual 3-dimensional maps of MD. Then, individual T1-weighted images were co-registered to the b0 images, using rigid-body transformation. These registrations were used to transform masks of the left and right anterior and posterior HC (derived from the T1 images) to the MD maps, for extraction of the mean individual hippocampal MD values. For this analysis FSL software was used.

To assess potential changes in RSFC of the HC, we used a customized processing stream based on the 1,000 Functional Connectomes Project (http://www.nitrc.org/projects/fcon_1000) (Biswal et al., 2010). Co-registered masks of the left and right anterior and posterior HC served as seeds for RSFC analysis, in line with previous studies (Rombouts et al., 2005; Andrews-Hanna et al., 2010; Witte et al., 2014). Pre-processing of individual functional scans comprised slice time correction, motion correction, spatial smoothing with a 6 mm full-width-half-maximum (FWHM) Gaussian kernel, temporal filtering (0.01–0.1 Hz), and de-trending, using AFNI and FSL software. The functional scans were normalized to the anatomical image, using affine co-registrations. Noise due to motion, white matter, cerebrospinal fluid, and global change was removed from the functional signal by multiple regressions, creating standardized residual BOLD-signal time series (in FSL). Then, mean time series of the individual HC seeds were correlated with times series of all other gray matter voxels in the brain, using a general linear model approach within FSL (FMRIB's local analysis of mixed effects with Ordinary Least Square option, FLAMEO). The resulting Pearson's r correlation coefficient 3D maps were then Fisher's z-transformed and smoothed, using a kernel of sigma = 1 to improve normality. This produced spatial maps in which the values of voxels represented the strength of the correlation with the individual HC seeds. Registration of individual z-maps for group analysis included a rigid body within-subject registration of both time points to a “halfway space” before affine and non-linear registrations to a study-specific template, as described in detail in previous studies (Witte et al., 2014; Köbe et al., 2016).

### SNP genotyping

DNA was extracted from whole blood, using a blood mini-kit (Qiagen, Hilden, Germany) and stored at −80°C until analysis. Genotyping of the single nucleotide polymorphisms (SNP) apolipoprotein E (APOE) rs429358 and rs7412 that have been previously implicated in cognitive performance (Corder et al., 1993; Egan et al., 2003; Witte and Floel, 2012) was performed, using a pre-designed Taqman assay at the laboratory of Prof. Dr. Dan Rujescu (University of Halle, Germany), following procedures described previously (O'Dwyer et al., 2012).

### Blood markers of glucose control, anthropometric measures and mood

After fasting overnight of at least 10 h, all subjects underwent venous blood sampling for assessment of serum levels of glucose, glycated hemoglobin A1c as long-term measure of glucose, insulin, high-to-low density lipoprotein (HDL-to-LDL) ratio, and high-sensitive C-reactive protein (hsCRP). All parameters were analyzed by IMD Laboratory, Berlin, Germany. Anthropometric measures included weight and body mass index (BMI). Patients also reported their physical activity and other lifestyle habits, using the Freiburger physical activity questionnaire (Frey et al., 1999). For mood ratings during neuropsychological testing, the Positive and Negative Affective Schedule (PANAS; Krohne et al., 1996) was used. Moreover, to characterize each patient's depression and anxiety level at baseline, the Beck's Depression Inventory (BDI; Kuhner et al., 2007) and the State-Trait Anxiety Inventory (STAI X1; Laux et al., 1981) were used.

### Statistical analyses

Before data analysis, all variables were tested for normal or near-normal distribution (unimodal, |skewness| <1). Accordingly, parametric and non-parametric tests were calculated, as appropriate. Two-sided level of significance was set at α < 0.05. SPSS 23.0 (PASW, SPSS; IBM, Armonk, NY) was used for the analysis.

At baseline, parameters of memory performance, glucose metabolism, HC volume and microstructure were compared between groups, using independent *t*-tests or Mann-Whitney *U*-tests, as appropriated. To detect differences between groups with regard to changes over time in these parameters, we performed repeated-measures analysis of variance (ANOVA_RM_) with “time” as repeated factor (baseline, follow-up) and “group” as between-subject factor (resveratrol, placebo intervention). Changes over intervention time within groups were evaluated, using paired *t*-tests or Wilcoxon signed-rank tests, as appropriate. Correction for multiple comparison was applied for our primary hypothesis that resveratrol intake has beneficial effects on memory performance in comparison to placebo intake, using Bonferroni threshold (α = 0.05 divided by the number of tests per category). Secondary hypotheses comprised detection of changes in glucose metabolism and HC RSFC and structure. For all secondary analyses no adjustment for multiple testing was applied.

HC seed-based RSFC group analysis included gray matter-voxelwise GLM statistics, implemented in FSL, between changes in HC RSFC in the resveratrol group compared with those in the placebo group, using a cerebral gray matter mask (cerebellum excluded). Gaussian random field theory was used to correct for multiple comparisons at the cluster level (FSL easythresh, clusterwise correction, *z* > 2.3, *p* < 0.05).

Initially all analyses were conducted unadjusted. Additionally, analyses with 40 available datasets (memory performance and glucose control) were corrected for 4 covariates (age, sex, APOE e4 carrier status, and education) and analyses with 30 available data sets (MRI analyses) were corrected for 3 covariates (age, sex, and APOE e4 carrier status), to allow adjustment for important covariates but to avoid over-fitting Stoltzfus (2011).

To detect associations between changes in glucose parameters, HC structure and RSFC, and memory performance after the 26-week intervention, we ran bivariate correlations; Pearson or Spearman's rank correlation analysis, according to distribution of the data.

## Results

### Baseline characteristics

At baseline, both intervention groups were similar with regard to sex, age, years of education, cardiovascular and psychological risk factors, standard markers for lipid and inflammatory status, physical activity and MMSE scores (Table 1). The placebo group comprised a higher number of APOE e4 carriers (59%) and a higher concentration of high-sensitive CRP (2,1 pg/ml) compared to the resveratrol group (28% and 1,3 pg/ml).

### Memory performance

Mean scores of all AVLT subtests, i.e., learning ability, delayed recall, retention and recognition, were comparable between intervention groups at baseline (see Table 2). Against our primary hypothesis no interaction effect of group × time was found for memory performance (ANOVA_RM_; all *d*s ≤ 0.471; *p* ≥ 0.157, Bonferroni corrected). Adjustment for age, sex, APOE e4-carrier status and education as well as a separate analysis for male and female patients did not change the results. For details see Table 2.

**Table 2:** **Intervention related changes in declarative memory performance of MCI patients dependent on group and time**.

	**Resveratrol (*n* = 18)**			**Placebo (*n* = 22)**				
	**BL**	**FU**	***p*-value**	**BL**	**FU**	***p*-value**	**Unadjusted *p*-value (group × time)^c^**	**Adjusted *p*-value (group × time)^d^**
Learning ability	44.9±9.5	43.0±10.7	0.314^a^	44.2±8.1	41.9±12.8	0.495^b^	0.878	0.840
Delayed recall	7.6±3.9	6.7±3.9	0.173^a^	7.2±2.7	7.6±3.7	0.536^a^	0.157	0.126
Retention	−3.4±2.1	−3.8±2.6	0.444^a^	−3.8±2.2	−3.3±2.2	0.505^a^	0.325	0.249
Recognition	9.8±5.9	8.4±6.2	0.122^b^	9.3±4.2	7.7±6.0	0.152^b^	0.882	0.806

*Number of words in different subtests of the auditory verbal learning test expressed as mean ± SD (raw data). BL, baseline; FU, follow-up*.

a *Paired t-test*.

b *Wilcoxon signed-rank test*.

c *ANOVA_RM_, unadjusted*.

d *ANCOVA_RM_, adjusted for age, sex, APOE e4 status and education*.

### Changes in parameters of glucose control

At baseline, parameters of glucose control were similar in both intervention groups (see Table 3).

**Table 3:** **Intervention related changes in fasting serum parameters of glucose metabolism of MCI patients dependent on group and time**.

	**Resveratrol (*n* = 18)**			**Placebo (*n* = 22)**				
	**BL**	**FU**	***p-*value**	**BL**	**FU**	***p*-value**	**Unadjusted *p*-value (group × time)^c^**	**Adjusted *p*-value (group × time)^d^**
HbA1c [%]^e^	5.86±0.36	5.71±0.33	**0.005^a^**	5.73±0.29	5.71±0.28	0.769^b^	0.059	0.066
Glucose [mg/dl]^f^	97.6±13.1	101.8±25.0	0.392^a^	96.7±15.2	97.2±15.1	0.807^b^	0.468	0.297
Insulin [mU]	8.7±3.7	10.4±12.6	0.349^b^	9.2±4.1	9.0±5.8	0.163^b^	0.521	0.402

*Data expressed as mean ± SD. Significant results are highlighted in bold (p < 0.05). BL, baseline; FU, follow-up; HbA1c, glycated hemoglobin A1c*.

a *Paired t-test*.

b *Wilcoxon signed-rank test*.

c *ANOVA_RM_, unadjusted*.

d *ANCOVA_RM_, adjusted for age, sex, APOE e4 status and education*.

e *Three subject excluded due to missing values (placebo group)*.

f *One subject excluded due to missing values (placebo group)*.

**Figure 2:**
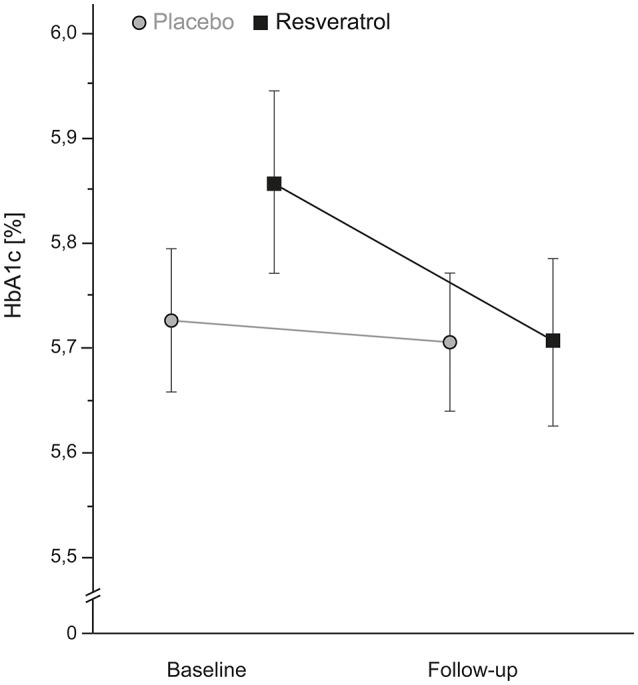
**A moderate, but non-significant decrease in the long-term glucose marker HbA1c after 26 weeks resveratrol intervention (*n* = 18) compared to placebo intervention (*n* = 19) (ANCOVA_RM_*p* = 0.059, *d* = 0.66)**. The decrease reached statistical significance when analyzing the resveratrol group separately (paired *t*-test *p* = 0.005). HbA1c, glycated hemoglobin A1c.

An interaction effect of group × time was found for HbA1c with a moderate effect size of *d* = 0.66 [ANOVA_RM_, *F*_(1, 35)_ = 3.80, *p* = 0.059], similar after full adjustment for age, sex, APOE e4 carrier status and education [ANCOVA_RM_, *F*_(1, 31)_ = 3.6, *p* = 0.066, *d* = 0.65]. Here, the long-term glucose marker HbA1c was significantly reduced in MCI patients after resveratrol intervention [−0.15%; paired *t*-test; *t*_(17)_ = 3.3, *p* = 0.005, *d* = 1.60]; this was not the case in the placebo group (−0.02%) (Figure 2). For glucose and insulin no group × time interaction effects and no changes over time in both groups were observed. For details see Table 3.

### Hippocampus resting-state functional connectivity

**Figure 3:**
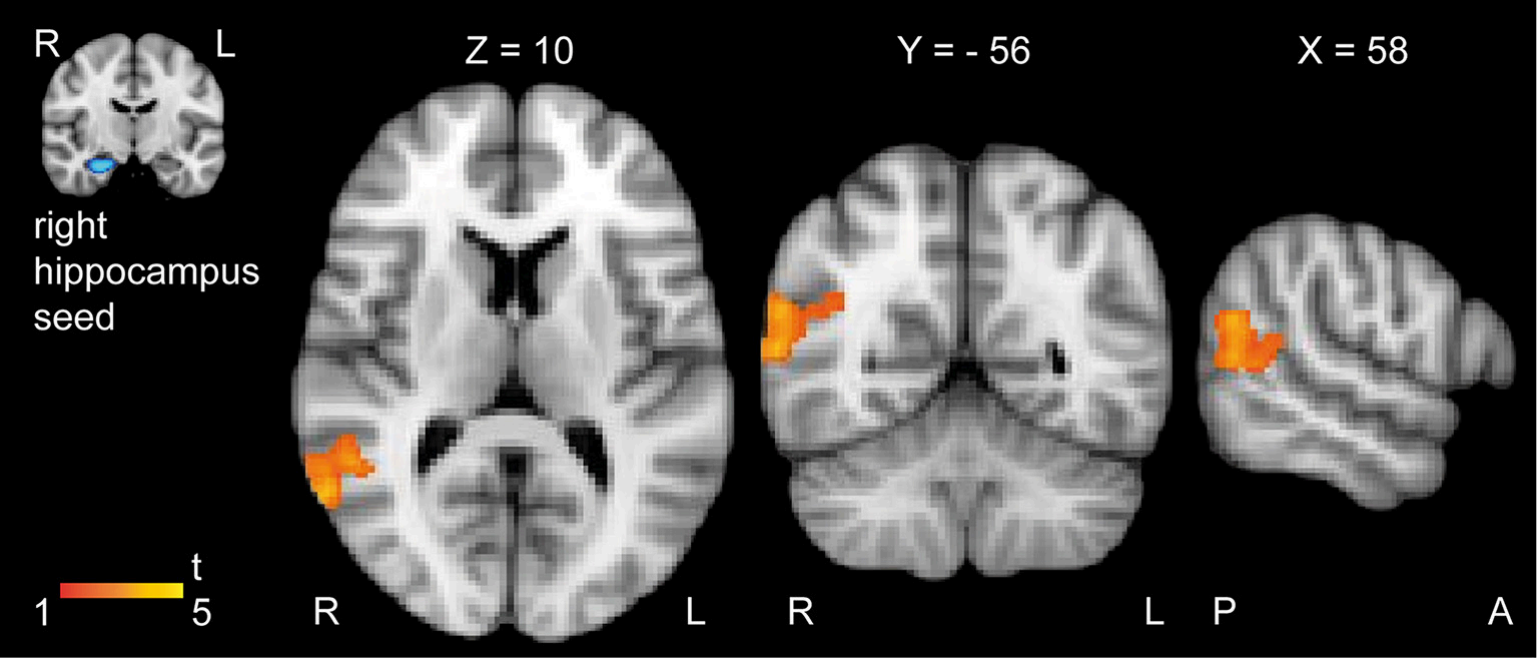
**Significant increase in resting-state functional connectivity between the right HC and the right angular cortex after 26 weeks resveratrol intervention (*n* = 14) compared to placebo intervention (*n* = 16)**. Color bar indicate *t*-values of significant voxels (resveratrol intervention > placebo intervention; cluster-based thresholding, *p* < 0.05). Individual masks of the left and right HC were used as seeds in the analysis. No group differences were observed for the opposite contrast (placebo intervention > resveratrol intervention), and when using the left HC as seed. For better visualization we superimposed the t-map on the MNI-template. Images are displayed in neurological convention, coordinates in mm according to MNI space. A, anterior; H, hippocampus; L, left; R, right; P, posterior.

The seed-based RSFC analysis revealed that resveratrol intervention significantly increased functional connectivity between the right HC seed and a cluster in the right angular cortex compared to placebo (Figure 3, yellow-red, cluster of 523 voxels, hot voxel: *x* = 60, *y* = −56, *z* = 10, *p* < 0.001). This effect was primarily driven by the anterior part of the right HC (cluster of 557 voxels, hot voxel: *x* = 60, *y* = −58, *z* = 10, *p* < 0.001). Results did not change after adjustment for age, sex and APOE e4 carrier status (*p* < 0.001) and after correction for multiple comparisons (FSL easythresh). The decrease in HbA1c did not significantly correlate with the increase in RSFC in the resveratrol group (*r* = −0.131, *p* = 0.665). We did not find significant group differences for the opposite contrast (placebo intervention > resveratrol intervention). No significant group differences were observed in correlations between the left HC seed and other gray matter brain regions.

### Gray matter volume, hippocampus volume, and microstructure

At baseline, total gray matter volume, and volume and microstructure of the HC were similar between both groups (see Table 4).

**Table 4:** **Intervention related changes in gray matter volume, and volume and microstructure of the hippocampus of MCI patients dependent on group and time**.

	**Resveratrol (*n* = 14)**			**Placebo (*n* = 16)**				
	**BL**	**FU**	***p*-value^a^**	**BL**	**FU**	***p*-value^a^**	**Unadjusted *p*-value (group × time)^b^**	**Adjusted *p*-value (group × time)^c^**
Total GM volume [cm^3^]	542.8±68.6	540.6±65.1	0.454	552.1±50.5	547.0±55.6	0.301	0.606	0.744
**HC, LEFT**								
Volume [cm^3^]	3.52±0.6	3.53±0.6	0.774	3.78±0.6	3.62±0.5	0.073	0.084	0.113
MD [m^2^/s]	1.10±0.19	1.12±0.14	0.629	1.14±0.09	1.14±0.11	0.736	0.544	0.131
**HC, LEFT ANT**								
Volume [cm^3^]	2.04±0.4	2.06±0.3	0.583	2.24±0.3	2.14±0.3	0.063	0.061	0.061
MD [m^2^/s]	1.16±0.16	1.15±0.17	0.850	1.17±0.10	1.17±0.13	0.686	0.899	0.380
**HC, LEFT POST**								
Volume [cm^3^]	1.47±0.2	1.47±0.3	0.863	1.55±0.2	1.48±0.2	0.133	0.222	0.340
MD [m^2^/s]	1.09±0.20	1.07±0.10	0.503	1.09±0.08	1.09±0.1	0.803	0.620	0.468
**HC, RIGHT**								
Volume [cm^3^]	3.71±0.5	3.75±0.5	0.463	3.82±0.6	3.72±0.6	0.257	0.183	0.107
MD [m^2^/s]	1.09±0.12	1.15±0.09	**0.029**	1.16±0.09	1.18±0.11	0.171	0.275	0.063
**HC, RIGHT ANT**								
Volume [cm^3^]	2.17±0.3	2.18±0.3	0.848	2.26±0.4	2.20±0.3	0.253	0.316	0.168
MD [m^2^/s]	1.16±0.10	1.20±0.11	**0.007**	1.21±0.12	1.23±12.8	0.266	0.374	0.429
**HC, RIGHT POST**								
Volume [cm^3^]	1.53±0.2	1.57±0.2	0.218	1.56±0.3	1.52±0.2	0.231	0.093	0.071
MD [m^2^/s]	1.04±0.08	1.06±0.09	0.073	1.09±0.07	1.12±0.1	0.252	1.000	0.453

*Number of words in different subtests of the auditory verbal learning test expressed as mean ± SD (raw data). Ant, anterior; BL, baseline; FU, follow-up; HC, hippocampus; MD, mean diffusivity; post, posterior*.

a *Paired t-test*.

b *ANOVA_RM_, unadjusted*.

c *ANCOVA_RM_, adjusted for age, sex, and APOE e4 status*.

*Significant results are highlighted in bold (p < 0.05)*.

**Figure 4:**
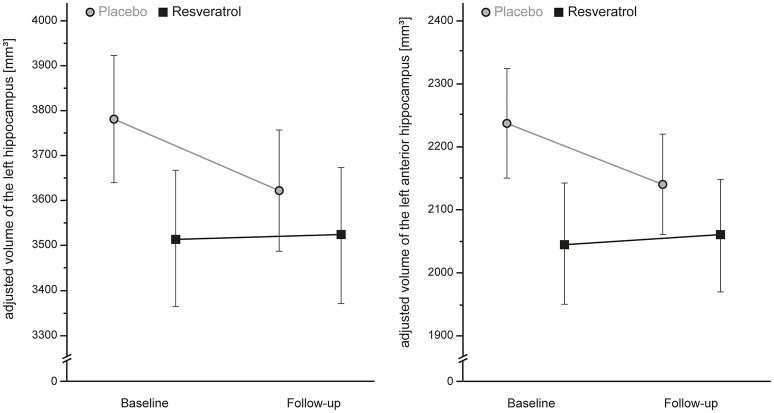
**A moderate, but non-significant, preservation of gray matter atrophy of the left, particularly anterior, hippocampus after 26 weeks resveratrol supplementation (*n* = 14) compared to placebo (*n* = 16) (ANCOVA_RM_; left Hippocampus, *p* = 0.084, *d* = 0.68; left anterior Hippocampus, *p* = 0.061, *d* = 0.74)**. Error bars indicate standard error.

At between-group level, total gray matter volume was similar after intervention period in both groups [ANCOVA_RM_, *F*_(28)_ = 0.272, *p* = 0.606, *d* = 0.20]. A group × time interaction effect was found for the atrophy rate of the left HC with a moderate effect size of *d* = 0.68 [ANCOVA_RM_; *F*_(28)_ = 3.2, *p* = 0.084], particularly for the left anterior HC [ANCOVA_RM_; *F*_(28)_ = 3.8, *p* = 0.061, *d* = 0.74] after no adjustment, and was still present after full adjustment for age, sex, and APOE e4 carrier status [ANCOVA_RM_; left HC, *F*_(24)_ = 2.3, *p* = 0.144, *d* = 0.57; left anterior HC, *F*_(24)_ = 3.2, *p* = 0.088, *d* = 0.68]. Patients of the placebo group showed a decrease of −4.2% in volume of the left, specifically left anterior (−4.5%) HC, whereas the volume was preserved in the resveratrol group (+0.3%). No group × time interaction effects were found for changes in volume of the left posterior HC and the right HC of both groups. For details see Table 4 and Figure 4.

Turning to HC microstructure, no selective differences were noted between groups for changes in mean diffusivity of the HC over time (see Table 4).

## Discussion

In this proof-of-concept study with MCI patients, we demonstrated that daily supplementation with 200 mg resveratrol over 26 weeks led to a moderate decrease in HbA1c (non-significant), a long-term marker of glucose control, in comparison to placebo. Moreover, supplementation significantly increased RSFC between the right HC and the right angular cortex and preserved the volume of the left, particularly anterior, HC with a moderate effect size (non-significant). Beneficial effects of resveratrol on memory performance and HC microstructure were not found.

The pathogenesis of “typical Alzheimer's disease” has been divided in sequential disease stages, characterized by changes in different biomarkers over time (Jack et al., 2013). According to this model, an initial increase in Aß deposition is followed by Tau pathology, hypometabolism, brain atrophy particularly of the HC, and finally cognitive dysfunction. Within this model, changes in the functional organization of the brain represent a promising diagnostic biomarker in early disease stages, preceding structural brain changes (Pievani et al., 2011). Notably, changes in RSFC are known to reflect dynamic modulations of blood flow and brain activity, well before structural alterations (Sheline et al., 2010; Prvulovic et al., 2011; Brickman et al., 2014), and may therefore constitute a highly sensitive biomarker to detect subtle changes due to dietary modifications. Moreover, it has been shown in previous studies that dietary changes can influence RSFC (Grayson et al., 2014; Witte et al., 2014; Wiesmann et al., 2016). A randomized controlled crossover study by Kennedy et al. (2010) demonstrated that a single dose of resveratrol was sufficient to increase cerebral blood flow, measured by near-infrared spectroscopy. However, long-term interventional studies that investigate the beneficial potential of nutritional components by measuring brain RSFC are still limited. In an own study with healthy older adults, resveratrol supplementation over 26 weeks compared to placebo significantly improved RSFC of the HC (Witte et al., 2014). Here, we were partially able to confirm these findings, i.e., found a significant increase in RSFC between the right HC and the right angular cortex in MCI patients, supporting the positive influence of resveratrol on brain networks even in early disease-stages. HC and angular gyrus are structurally and functionally connected (Uddin et al., 2010), and constitute core areas within the default mode network, known to be related to memory function (McCormick et al., 2014) and to deteriorate in aging and neurodegenerative disease (Dennis and Thompson, 2014).

In addition, resveratrol supplementation showed a moderate effect (non-significant) on preservation of hippocampal volume, which was decreased over time in the placebo group. This result is in line with a reduction of neurodegeneration and/or an increase in neurogenesis of the HC observed after resveratrol injection in rodent models, even in older age (Kim et al., 2007; Kodali et al., 2015). In our previous resveratrol trial with healthy older adults, we could not demonstrate a beneficial effect of resveratrol on HC structure. However, the specific cohort in that study, i.e., healthy older adults, might have prevented us from observing significant differences, due to lower atrophy rates over the course of 6 months in the healthy group (Witte et al., 2014). MCI patients show a stronger atrophy rate within 6 months compared to healthy older adults (McDonald et al., 2009), which might increase the probability for statistical detection of sensitive dietary effects on changes in brain morphology. Given that we only found a moderate and non-significant effect of resveratrol on HC structure, longer intervention times may be necessary to induce significant changes (Douaud et al., 2013).

So far, studies on the impact of resveratrol on cognitive performance showed mixed results. First interventional studies have shown that resveratrol supplementation over 18 and 6 months, respectively, improved spatial memory performance in non-human primates (Dal-Pan et al., 2011), and increased the number of words retained over 30 min in healthy older adults (Witte et al., 2014). In contrast, in a recently published randomized-controlled trial, resveratrol intervention over 28 days was not sufficient to induce clear improvements in cognitive function, showing merely improvements in working memory performance (i.e., accuracy in the 3-back task), but not in tasks related to attention and executive function (Wightman et al., 2015). In the current study, we did not detect resveratrol-related beneficial effects on learning and memory performance in MCI patients, possibly due to the small sample size and still relatively short intervention time [6 months as compared to 24 months in a nutritional study with B-vitamin supplementation that demonstrated improvement in episodic memory performance (de Jager et al., 2012)]. Thus, our study design may have only allowed for changes in RSFC and moderate effects on structure but not yet for “downstream” cognitive effects.

Neuroprotective mechanisms that might explain the link between resveratrol and improved brain structure, and eventually function, include reductions in mitochondrial dysfunction, oxidative damage, glucose toxicity, and chronic inflammation, by improving glucose metabolism and vascular functions, see also Huhn et al. (2015) for detailed review. Moreover, resveratrol is described as a potential activator of the sirtuin pathway that is regulated by NAD+/NADH, linking energy metabolism to gene expression (Timmers et al., 2011; Li, 2013). Our previous study in healthy older adults demonstrated a significant decrease in the long-term glucose marker HbA1c after resveratrol intervention compared to placebo (Witte et al., 2014). In line with these findings, we also found a moderate, but non-significant, reduction in HbA1c after 26 weeks resveratrol treatment in MCI patients in comparison to an unchanged value after placebo intake. These findings point toward resveratrol-induced modulations of glucose control as one possible mechanism underlying beneficial effects on RSFC (Stranahan and Mattson, 2008).

Some limitations should be considered when interpreting our findings. First, the small number of patients in both groups might have prevented us from observing statistically significant changes in memory function and secondary outcome parameters, i.e., HbA1c, and HC volume and microstructure. Second, an intervention time of 26 weeks might be too short to reach significant beneficial effects. Third, intervention and placebo group differed in the number of APOE e4 carriers, which might have biased the results; however, adjustment for APOE e4 status did not attenuate the moderate (non-significant) effects. Fourth, self-reported information by the patients and count of remaining capsules were the only compliance measure, but group-specific decrease in HbA1c supports patients' adherence. Fifth, resveratrol was given in a formula with quercetin to increase its bioavailability (De Santi et al., 2000), whereby an impact of quercetin itself on brain function cannot be excluded. However, in a randomized-controlled trial even higher doses of quercetin (500 mg/d or 1,000 mg/d) did not show ergogenic effects on neurocognitive functioning in humans (Broman-Fulks et al., 2012).

In sum, we were able to partially translate previously reported beneficial effects of resveratrol from healthy older adults to at-risk patients for Alzheimer's disease, showing significantly increased HC RSFC, and moderate improvements in glucose metabolism and HC structure (non-significant). We believe that these findings support resveratrol as a potential non-pharmacological agent to modify the disease process in MCI patients. However, larger and long-term interventional trials in patients are now needed to confirm or refute beneficial effects observed on sensitive surrogate markers for hippocampal function, and to determine if these findings also extend to cognitive function.

## Author contributions

Substantial contributions to the conception or design of the work: AW and AF. Data acquisition: TK, AS, VT, JP, JS, AH, and JB. Data analyses: TK, AW, and UG. Data interpretation and discussion: TK, AW, AS, VT, JP, JS, AH, and AF. Manuscript drafting: TK, AW, and AF. Revising Manuscript critically for important intellectual content: TK, AW, AS, VT, JP, JS, AH, JB, UG, and AF. Final approval of the version to be published: TK and AF.

### Conflict of interest statement

The authors declare that the research was conducted in the absence of any commercial or financial relationships that could be construed as a potential conflict of interest.
